# The relationship between the severity of disability and serum IL-8 in acute ischemic stroke patients

**DOI:** 10.1186/s41983-018-0025-z

**Published:** 2018-09-24

**Authors:** Hala A. Shaheen, Lamiaa I. Daker, Mohammed M. Abbass, Asmaa A. Abd El Fattah

**Affiliations:** 1 Department of Neurology, Faculty of Medicine, Fayoum University, PO Box: 63514, Fayoum, Egypt; 2 Department of Clinical Pathology, Faculty of Medicine, Fayoum University, Fayoum, Egypt

**Keywords:** Acute ischemic stroke, Severity of disability, IL-8

## Abstract

**Background:**

Stroke is the third leading cause of death and leading cause of adult disability worldwide. Long-term disability is a significant problem among survivors; post-stroke inflammation is well known to contribute to the expansion of the ischemic lesion resulting in significant morbidity and disability. To study the impact of serum level of IL-8 on severity of disability in patients with acute ischemic stroke in the first 48 h post stroke.

**Methods:**

A cross-sectional case control study was conducted on 44 patients with acute ischemic stroke (in the first 48 h). The patients were subjected to full neurological examination, computed tomography (CT) and magnetic resonance imaging (MRI) of the brain, and assessment of stroke disability using the National Institute of Health Stroke Scale (NIHSS) and modified Rankin Scale (mRS). Measurement of the serum levels of IL-8, erythrocyte sedimentation rate, and C-reactive protein (CRP) was done. Forty-four matched control subjects for their age and sex were included for comparison of serum level of IL-8.

**Results:**

The level of IL-8 was significantly higher in the patients than in the control subjects (*p* < 0.001).There was a statistically significant positive correlation between serum level of IL-8 and disability assessed by NIHSS (*r* = 0.42, *p* = 0.004). The patients with moderate disability showed significant higher IL-8 levels than those with minor disability (*p* = 0.02).

**Conclusion:**

The severity of disability in early acute ischemic stroke is highly correlated to the serum level of IL-8.

## Background

Stroke is the third leading cause of death and leading cause of adult disability worldwide. Several studies had shown that nearly 15–30% of stroke survivors are permanently disabled, and 20% of stroke survivors require institutional care 3 months following stroke (Lloyd-Jones et al. 2010). As the burden of disability following stroke is unpredictable, a need exists to identify the causative biomarkers so that individualized post-ischemic stroke treatment regimens could be developed (VanGilder et al. 2014).

Few studies have investigated the relationship between acute biomarkers and functional outcome following stroke. It had been shown that a time course of inflammatory cytokines (i.e., interleukin-2, interleukin-10, and tumor necrosis factor-α) can predict outcome. It was suggested that the post-stroke immune response occurs in a time-dependent fashion with the innate immune response occurring in the first hours following ischemic injury (Yan et al. 2012). Several studies have reported that higher levels of inflammatory markers such as C-reactive protein (CRP), interleukin-8, and interleukin-6 are associated with worse outcome after the ischemic strokes (Whiteley et al. 2009). This study aimed to evaluate the impact of the serum level of IL-8 on disability in patients with acute ischemic stroke in the first 48 h post stroke.

## Methods

This study is a cross-sectional case control study conducted on 44 patients of both sexes with diagnosis of acute ischemic stroke (within the first 48 h). They were recruited from the Neurology Outpatient Clinic and Emergency Department, Fayoum University, from October 2015 to September 2016. Written informed consent was obtained from all patients and the control groups. The age of patients ranged from 21 to 71 years old.

The patients who presented with hemorrhagic stroke were excluded from the study. Patients who presented with significant acute medical illness such as renal, hepatic, and autoimmune diseases resulting in elevated erythrocyte sedimentation rate (ESR) and interleukin levels and patients with history of cancer or receiving immunosuppressant drugs were also excluded from the study.

Forty-four (age, sex, and educational level) matched healthy controls were selected as a control group for comparison of the serum IL-8 level. They had no neurological disorders or any vascular risk factor.

All the patients underwent the following battery of evaluation of severity of disability by using the National Institute of Health Stroke Scale (NIHSS) and Modified Rankin Scale (mRS). Routine biochemical tests as CBC, ESR, liver functions, kidney functions, and blood sugar were done to exclude patients with systemic or metabolic disorders. Total cholesterol, low-density lipoprotein cholesterol (LDL-C), high-density lipoprotein cholesterol (HDL-C), and triglyceride levels were withdrawn from the patients and controls after fasting (at least 10 h) and sera were separated. Autoanalyzer by colorimetric method was applied for triglycerides and cholesterol measurements (NZOKIT Ranbaxy).

Serum level of interleukin 8 (IL-8) was obtained within the first 48 h of the ischemic stroke before starting the anti- ischemic management. Suspensions of serum were centrifuged within 30 min at 1500*g* for 10 min and immediately frozen and stored at − 80 °C until analysis. The serum IL-8 values were measured using enzyme-linked immunoabsorbent assay (ELISA) method according to the manufacturer’s instructions (Iłzecka and Stelmasiak n.d.). Serum CRP level was measured using ELISA with a normal value below 6 mg/dl.

Computed tomography (CT) or magnetic resonance imaging (MRI) 1.5 T (diffusion-weighted image) of the brain was done for all patients in the Radiology Department in Fayoum University Hospital to assess the size and location of the ischemic infarction.

### Statistical analysis

Statistical Package for Social Sciences (SPSS) version 18 was used for data management and analysis. Qualitative data were presented as numbers and percentages, quantitative data were presented as arithmetic means, and central tendency measurement and standard deviations were presented as measures of dispersion. The Mann-Whitney test was used for comparison of qualitative variables, while Spearman’s test was used for correlations. Chi-square test was used to compare qualitative variables and bivariate Pearson correlation test to test association between the different variables. The level *p* < 0.05 was considered the cut-off value for significance.

## Results

**Fig. 1:**
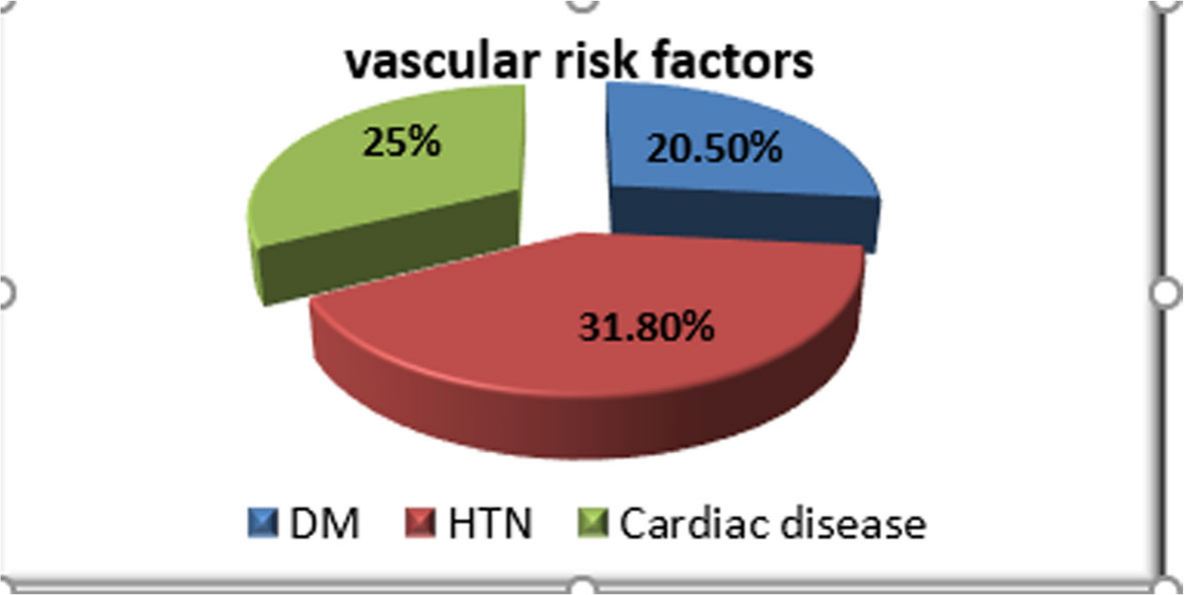
Distribution of vascular risk factors among the patient groups

The mean of age of patients was 0.02 ± 14.9 and controls 47.7 ± 15.7. The percentage of sex distribution (male to female %) was 61.4:38.6 for patients and 59.1:40.9 for controls. There was no significant difference in age and sex (*p* = 0.1, *p* = 0.9, respectively). Clinical characteristics of the patients are as follows: 36 patients (81.8%) presented with manifestation of anterior circulation ischemia and eight patients (18.2%) presented with manifestation of posterior circulation ischemia according to Oxfordshire Community Stroke Project (OCSP) classification (Iłzecka and Stelmasiak n.d.). The distribution of the vascular risk factors among the patients is shown in Fig. 1.

The mean National Institutes of Health Stroke Scale (NIHSS) score was 4.3 ± 2.1. Twenty-two (50%) patients had minor stroke and 22 (50%) patients had moderate stroke. The mean Modified Rankin Scale (mRS) score was 1.8 ± 0.86. Twenty-three patients had no significant disability (52.3%), nine patients had slight disability (20.5%), and 12 patients had moderate disability (27.3%).

**Fig. 2:**
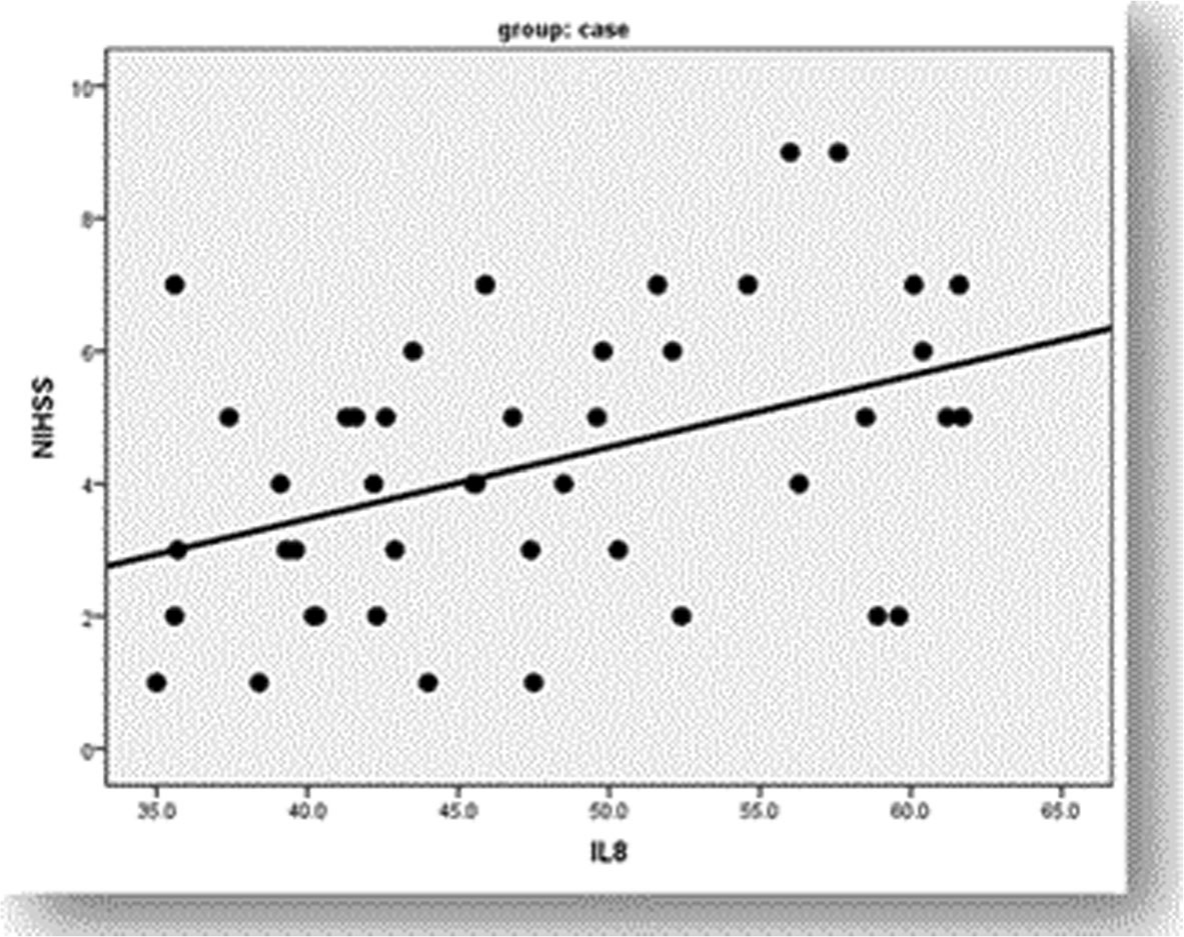
Correlation between IL-8 serum level and disability of stroke

It was shown that the patients had a significantly higher serum level of IL-8 (47.6 ± 8.3) than the controls (14.6 ± 5.9) (< 0.001). The patients with moderate disability assessed by NIHSS showed significant higher serum IL-8 level than those with minor disability (*p* = 0.02) as shown in Table 1. Moreover, there was a statistically significant positive correlation between IL-8 and NIHSS scores (*r* = 0.42, *p* = 0.004) as shown in Fig. 2. On the other side, there was no significant difference between those assessed by mRS as regards IL-8 as shown in Table 1, and there was no significant correlation between IL-8 and mRS scores (*r* = 0.19, *p* = 0.2).

**Table 1:** Comparison between the different grades of disability as regards serum IL-8

Stroke disability		IL-8 mean ± SD	*p* value
mRS	No disability	45.7 ± 7.6	0.2
	Slight disability	51.3 ± 7.8	
	Moderate disability	48.7 ± 9.5	
NIHSS	Minor disability	44.8 ± 7.2	0.02
	Moderate stroke	50.4 ± 8.5

As regards the neuroimaging of the brain, there was no significant difference in the serum level of IL-8 as regards the localization and lateralization of lesions as shown in Table 2.

**Table 2:** Comparison of the serum level of IL-8 in different radiological characters

Neuroradiological data (no. of patients)		IL-8		*p* value
		Mean ± SD		
Localization	Supra-tentorial (36)	48.3 ± 9.7		0.8
	Infra-tentorial (8)	47.5 ± 8.2		
Lateralization	Right (25)	50.1	7.6	0.08
	Left (19)	45.7	8.5	

There was no significant difference in NIHSS or mRS scores between the patients as regards ESR and CRP as shown in Tables 3 and 4.

**Table 3:** Comparison of the serum level of ESR in different disability scales

Stroke disability		ESR	*p* value
		Mean ± SD	
mRS	No significant disability	35.9 ± 19.8	0.2
	Slight disability	24 ± 27.7	
	Moderate disability	44.9 ± 31	
NIHSS	Minor stroke	30.3 ± 20.3	0.1
	Moderate stroke	41.6 ± 28.9

**Table 4:** Comparison of CRP level in different disability scales

Disability scales		CRP				*p* value
		Negative		Positive		
		No. of patients	%	No. of patients.	%	
mRS	No significant disability	6	54.5%	17	51.5%	0.2
	Slight disability	4	36.4%	5	15.2%	
	Moderate disability	1	9.1%	11	33.3%	
NIHSS	Minor stroke	6	54.5%	16	48.5%	0.9
	Moderate stroke	5	45.5%	17	51.5%	

## Discussion

Stroke is a major cause of hospitalization, chronic disability, and death. There is increasing evidence that ischemic brain injury secondary to arterial occlusion is characterized by acute local inflammation. During reperfusion after acute ischemia, polymorphonuclear neutrophils are believed to exacerbate tissue damage by both physical obstruction of vessels and release of oxygen radicals; proinflammatory cytokines, including interleukin (IL)-1b, IL-6, IL-8, IL-10, and TNF-a; and granulocyte-macrophage colony-stimulating factor (Kostulas et al. 1998).

In this study, the patients with acute ischemic stroke showed a significant higher level of IL-8 in the first 48 h post stroke than controls. This was in agreement with previous studies (Kostulas et al. 1998; Domac and Misirli 2008). Moreover, Kostulas et al. (1998) stated that increased concentrations of IL-8 can be detected intrathecally in patients with ischemic stroke. However, it was not restricted to the CNS. This upregulation of IL-8 mRNA expression occurred early within the first few days after onset of symptoms and remained elevated up to 1 month.

It was postulated that IL-8 is a neutrophil chemotaxic and activating factor as it stimulates the binding of neutrophils to the receptors which causes a conformational change in the integrin molecules, and facilitates adhesion and transmigration (Adams and Lloyd 1997). It has been clearly shown that local expression of IL-8 in the ischemic brain establishes a concentration gradient over the blood-brain barrier, and as a result of movement of migrating cells toward this gradient, the neutrophils rapidly penetrate into the brain parenchyma and cause local inflammation (Yamasaki et al. 1995).

In this study, the patients with moderate disability assessed by NIHSS showed significantly higher serum IL-8 level than those with minor disability as well as there was a significant positive correlation between NIHSS and serum IL-8 level which was in agreement with other previous studies (Chernykh et al. 2016; Li et al. 2015). Chernykh et al. (2016) stated a significant positive correlation of stroke severity with production of IL-8 which was not influenced by the duration of post-stroke period. These findings could be explained by that the serum level of IL-8 is corresponding to the extent of cerebral ischemia, cortical damage, and the resulting disability (Li et al. 2015).

On the other side, there was no significant difference between patients with no, slight, and moderate disability assessed by mRS as regards IL-8 in this study. This was in contrast to others (Domac and Misirli 2008) who found higher serum of IL-8 in the patient with higher disability (mRS scores ≥ 3). This contradiction between NIHSS and mRS results could be explained by that 52% of the patients had no disability on mRS scale and 20% had moderate disability versus about 50% of the patients had moderate disability on NIHSS. Moreover, the NIHSS appeared more sensitive than mRS in evaluation disability in the acute stage of ischemic stroke (Roberts et al. 1998).

Several studies have also focused on biomarkers to predict stroke outcome and treatment response, which can be completely different between patients. Stroke prognosis might also influence medical decisions about sending stroke patients to specialized stroke units, palliative care, and rehabilitation programs or deciding the best moment for discharge (Simats et al. 2016).

Despite the fact that most of these inflammatory biomarkers are not specific to ischemic stroke, the levels of several inflammatory mediators correlate with stroke severity and outcome. Inflammatory biomarkers such as CRP or several pro-inflammatory cytokines, especially IL-6, have been widely associated with poor functional outcome after cerebral ischemic events (Counsell and Dennis 2001).

C-reactive protein (CRP) is a biomarker of inflammation and may reflect progression of vascular disease. Conflicting evidences suggested CRP may be a prognostic biomarker of ischemic stroke outcome. Most studies that have examined the relationship between CRP and ischemic stroke outcome have used mortality or subsequent vascular events as the primary outcome measure (VanGilder et al. 2014). A recently published prospective case-control study also reported that an elevated CRP level at admission was an independent predictor of functional outcome in the first month following acute ischemic stroke (Abubakar et al. 2013).

It was postulated that estimation of proinflammatory serum biomarkers such as high-sensitivity CRP (IL-6) in acute stroke would be highly beneficial in assessing the care pathway for patients and their treatment options, as the most important challenge facing physicians is to reduce the unacceptable burden of stroke (Bharosay et al. 2011). However, there was no significant difference in disability assessed by NIHSS or mRS between the patients with positive and negative CRP in this study. In agreement with this result, Idicula et al. (2009) had suggested no association between high CRP level at admission and poor outcome. The lack of association might be attributed to the short outcome period (in the first few days).

Another explanation for lack of correlation could be due to measurement of low-sensitivity CRP in the patients of this study in spite of high-sensitivity CRP (hs-CRP) assay, this could not be sufficiently sensitive to measure blood levels of CRP within the normal range (< 10 mg/L); however, the development of high-sensitivity assays for CRP (hs-CRP) had permitted detection of even mild elevation of CRP, even within the normal range (Roberts et al. 2000).

The limitation of this study included lack of measurement of body mass index (BMI) as an indicator of obesity, and its correlation with the serum level of IL-8 as a major risk factor of ischemic stroke should be done in further studies.

## Conclusions

It was concluded that the severity of disability in early acute ischemic stroke (first 48 h post stroke) is highly correlated to the serum level of IL-8.

## Abbreviation

CNS: Central nervous system
CRP: C-reactive protein
CT: Computed tomography
ELISA: Enzyme-linked immunoabsorbent assay
ESR: Erythrocyte sedimentation rate
HDL-C: High-density lipoprotein cholesterol
IL: Interleukin
LDL-C: Low-density lipoprotein cholesterol
MRI: Magnetic resonance imaging
mRS: Modified Rankin Scale
NIHSS: National Institute of Health Stroke Scale
OCSP: Oxfordshire Community Stroke Project
SPSS: Statistical Package for Social Sciences
